# Scalable expansion of human pluripotent stem cells under suspension culture condition with human platelet lysate supplementation

**DOI:** 10.3389/fcell.2023.1280682

**Published:** 2023-10-12

**Authors:** Haitao Yuan, Hong Su, Chen Wu, Yibing Ji, Lili Zhou, Lingna Wang, Haihong Zhang, Xin Zhang, Xiaopeng Tian, Fangfang Zhu

**Affiliations:** ^1^ School of Biomedical Engineering, Shanghai Jiao Tong University, Shanghai, China; ^2^ HemaCell Biotechnology Inc., Suzhou, China; ^3^ National Clinical Research Center for Hematologic Diseases, Jiangsu Institute of Hematology, The First Affiliated Hospital of Soochow University, Suzhou, China; ^4^ Institute of Blood and Marrow Transplantation, Collaborative Innovation Center of Hematology, Soochow University, Suzhou, China

**Keywords:** human platelet lysate, human pluripotent stem cell, suspension culture, serum free, cell proliferation

## Abstract

The large-scale production of human pluripotent stem cells (hPSCs), including both embryonic stem cells (hESCs) and induced pluripotent stem cells (hiPSCs), shows potential for advancing the translational realization of hPSC technology. Among multiple cell culture methods, suspension culture, also known as three-dimensional (3D) culture, stands out as a promising method to fulfill the large-scale production requirements. Under this 3D culture condition, cell expansion and the preservation of pluripotency and identity during long-term culture heavily relies on the culture medium. However, the xenogeneic supplements in culture medium remains an obstacle for the translation of cell and gene therapy applications from bench to bedside. Here, we tested human platelet lysate (hPL), a xeno-free and serum-free biological material, as a supplement in the 3D culture of hPSCs. We observed reduced intercellular variability and enhanced proliferation in both hESC and hiPSC lines. These cells, after extended culture in the hPL-supplemented system, maintained pluripotency marker expression, the capacity to differentiate into cells of all three germ layers, and normal karyotype, confirming the practicability and safety of hPL supplementation. Furthermore, through RNA-sequencing analysis, we found an upregulation of genes associated with cell cycle regulations in hPL-treated cells, consistent with the improved cellular division efficiency. Taken together, our findings underscore the potential of hPL as a xeno-free and serum-free supplement for the large-scale production of hPSCs, which holds promise for advancing clinical applications of these cells.

## 1 Introduction

hPSCs, including hESCs and hiPSCs, constitute vital cellular resources with immense potential in regenerative medicine and pharmaceutical research. These cells have been extensively utilized in drug discovery (Vandana et al., 2023), *in vitro* disease modeling (Sharkis et al., 2012), and cell-based therapies (Yamanaka, 2020). However, current protocols for cultivating hPSCs on a laboratory scale fall short of fulfilling these rigorous demands. To illustrate, addressing heart repair post-myocardial infarction or treating type 1 diabetes through β-cell replacement would require a minimum of 1–2 × 10^9 specialized cells to provide treatment for a single patient (Kropp et al., 2017). Thus, the imperative for large-scale hPSC production becomes crucial to meet the requirements of clinical applications and drug discovery.

Over the last few decades, multiple cell culture protocols have been explored for hPSCs. Among these, suspension culture, also known as 3D culture, has emerged as a promising large-scale production method to fulfill the substantial hPSC quantity requirements outlined above (Chen et al., 2014). The 3D culture of hPSCs includes expansion strategies based on micro-carriers or aggregates (Chen et al., 2014). Unlike the microcarrier culture system, the aggregated culture system eliminates the need to remove microcarriers from cell preparations before their applications. Additionally, the matrix-free, cell-only aggregates formed in the aggregated culture system hold the potential to closely mimic the “*in situ* niche” of hESCs, resembling the inner cell mass of early human embryo at the blastocyst stage (Blakeley et al., 2015).

While this aggregated culture system has been explored to enhance the large-scale production of hPSCs, the culture medium remains a central focus to improve the yield and reproducibility across different hPSC lines in a GMP-compliant setting (Liu et al., 2019). Basal culture media, such as DMEM/F12, can supply the most of medium nutrients, salts and vitamins. Nevertheless, the addition of supplementary components such as fetal bovine serum (FBS), or defined mixture like knockout serum replacement (KOSR) and B27 supplement, is commonly necessary. However, FBS contains undefined components with lot-to-lot variations, KOSR was originally designed to fit the feeder cell-based culture, and even B27 has bovine serum albumin (BSA) as a major component (Brewer et al., 1993). The significant presence of any primary and secondary raw materials derived from non-human animals in the manufacturing processes would challenge the translation from bench to bedside in cell and gene therapy applications. Hence, the development of xeno-free supplement to improve culture system is desirable. From this standpoint, we identified a suitable supplement, hPL, which holds the potential in improving the efficiency and consistency of the hPSC culture system.

hPL is a xeno-free and serum-free biological material derived from human platelets. It has been shown to support the growth of various cell types including mesenchymal stem cells (MSCs), endothelial cells, and osteocytes (Hofbauer et al., 2014; Iudicone et al., 2014; Shanbhag et al., 2022). Notably, hPL supplementation has been shown to not only improve cell proliferation but also maintain the stem cell markers of MSCs (Iudicone et al., 2014; Mareschi et al., 2022). Furthermore, in stem cell differentiation, such as hematopoietic stem cells, hPL has exhibited performance comparable or even superior to that of human AB serum (Li et al., 2023). Despite its potential as a xeno-free, serum-free supplement for stem cell production under GMP conditions, hPL supplementation has not yet been explored in the cultivation of hPSCs.

In our study, we introduced hPL into the 3D aggregated culture system and observed its positive impact on cell proliferation and viability. Notably, in the hPL-supplemented system, hPSCs maintained stem cell characteristics and normal karyotype after continuous passaging. RNA-seq analysis revealed significant alterations in the gene expression associated with cell cycle regulation and stem cell proliferation in hPL-treated cells. These findings indicate the promise of hPL as a xeno-free, serum-free supplement for large-scale stem cell production under GMP conditions.

## 2 Materials and methods

### 2.1 Cell culture

H1 hESCs were kindly provided by Dr. Donghui Zhang (Hubei University, Wuhan, China), and the peripheral blood mononuclear cells (PBMCs)-derived hiPSCs were purchased from CELLAPY Bio (Beijing, China). Cells were cultured using the mTeSR™ 1 Complete Kit (STEMCELL, 85,850) in a shaking incubator set at 80rpm, at 37°C with 5% CO2. The hPL-treated group was supplemented with 0.3% hPL (SETXON, PL-NH-100). Passaging is performed every three to 4 days. To passage the hPSCs, cell aggregates were collected, centrifuged at 100 g for 3 min, incubated with ACCUTASE (STEMCELL, 07,920) at 37°C, and dissociated into single cells. Cells were then centrifuged at 300 g for 5 min, and resuspended in mTeSR™ 1 medium with 10 μM Y-27632 (Sellseck, S1049) in the presence or absence of hPL. Cells were seeded at the density of 10^5 cells/mL in 6 well cell culture plates. Daily half medium change was performed until passaging.

### 2.2 Cell viability assay

Cell viability was determined with the CCK-8 assay. One day after passaging, hPL-treated and hPL-free cell aggregates were collected and centrifuge at 100 g for 3 min. Cells were then resuspended in culture medium containing 10% CCK-8 (Solarbio, CA1210-100) and incubated at 37°C for 4 h. Absorbance at 450 nm was then measured.

### 2.3 Cell cycling analysis

10^6 single cells were collected, washed twice with 1 mL pre-cooled PBS, and then resuspended in 300 μL PBS. After that, 700 μL pre-cooled absolute ethanol was added to fix the cells at 4°C overnight. The next day, cells were washed with PBS, and incubated with 250 μL PI staining solution (containing RNase) at room temperature in the dark for 15 min. Then, flow cytometry analysis was performed on LSRFortessa X-20 (BD Biosciences).

### 2.4 Flow cytometry analysis

Cell aggregates were dissociated with ACCUTASE (STEMCELL, 07,920), wash once, resuspended in 450 uL FACS buffer (PBS with 1% FBS and 8 μM EDTA). Then 100ul FACS buffer containing antibodies was added to each sample and incubated on ice for 20 min. After incubation, cells were washed twice, and resuspended in 200 ul FACS buffer containing DAPI. Flow cytometry was performed on LSRFortessa X-20 (BD Biosciences), and all flow cytometry data were analyzed with FlowJo (Tree Star).

To analyze the stem cell markers of the cultured cells, we used the following antibodies purchased from Biolegend: FITC anti-human SSEA-4 Antibody; PE anti-human TRA-1-60 Antibody; FITC Mouse IgG3, Isotype Ctrl Antibody; PE Mouse IgM, Isotype Ctrl Antibody.

### 2.5 Karyotype analysis

The cells were cultured to the 10th passage and then sent to Feifan Testing Co., Ltd, for chromosome G-banding analysis.

### 2.6 Trilineage differentiation analysis

The cultured cells were evaluated for their trilineage (ectoderm, mesoderm, and endoderm) differentiation potential utilizing the STEMdiff™ Trilineage Differentiation Kit (STEMCELL, 05230). Cells from both the fourth and 10th passages were chosen for the analysis, following the manufacturer’s instructions.

### 2.7 Immunofluorescence staining assay

5000 cells were transferred to one well of a 24-well plate pre-coated with Matrigel (Corning, 354277). After 2–3 days, cells were fixed with 4% paraformaldehyde at 4°C for 10 min, washed twice with PBS, permeabilized with 0.1% Triton X-100 for 10 min and blocked with PBS containing 1% FBS for 30 min. The cells were then incubated with primary antibodies added at a 500-fold dilution at 4°C overnight. After several washes with PBS, cells were incubated with secondary antibodies added at a 500-fold dilution at room temperature in the dark for 1 h. Cells were finally incubated with DAPI at room temperature for 10 min, and observed under a fluorescence microscope.

To analyze the stem cell markers of the cultured cells, we used the following primary antibodies (all purchased from Abcam): Anti-SOX2 antibody (ab92494); Anti-OCT4 antibody (ab181557); Anti-TRA-1-60 antibody (ab16288); Anti-SSEA4 antibody (ab16287); Anti-NANOG antibody (ab109250). Secondary antibodies were purchased from Thermo Fisher Scientific: Donkey anti-Mouse IgG (A32744); Donkey anti-Rabbit IgG (A32754); Goat anti-Mouse IgG (A32723); Goat anti-Rabbit IgG (A32731).

### 2.8 RNA-sequencing analysis

RNA was extracted from the fourth generation of cell aggregates and sent to Suzhou GENEWIZ Technology Co., Ltd for RNA-sequencing analysis. QC assessment was performed by FastQC (V0.10.1). Different expression gene analysis was performed by DESeq2 (V1.6.3). GO enrichment analysis was performed by TopGO (V2.18.0).

The RNA-seq dataset generated during this study has been deposited to NCBI Trace Archive NCBI Sequence Read Archive, this data can be found here: https://www.ncbi.nlm.nih.gov/sra/PRJNA1019100.

### 2.9 Statistical analysis

Data are presented as the mean plus or minus standard deviation. The significance of differences between groups was determined by a two-tailed unpaired *t*-test. *p*-values <0.05 were considered statistically significant. Statistical analysis was performed with GraphPad Prism 9.

## 3 Results

### 3.1 Enhanced viability and proliferation in hPL-supplemented 3D culture of hPSCs

**FIGURE 1:**
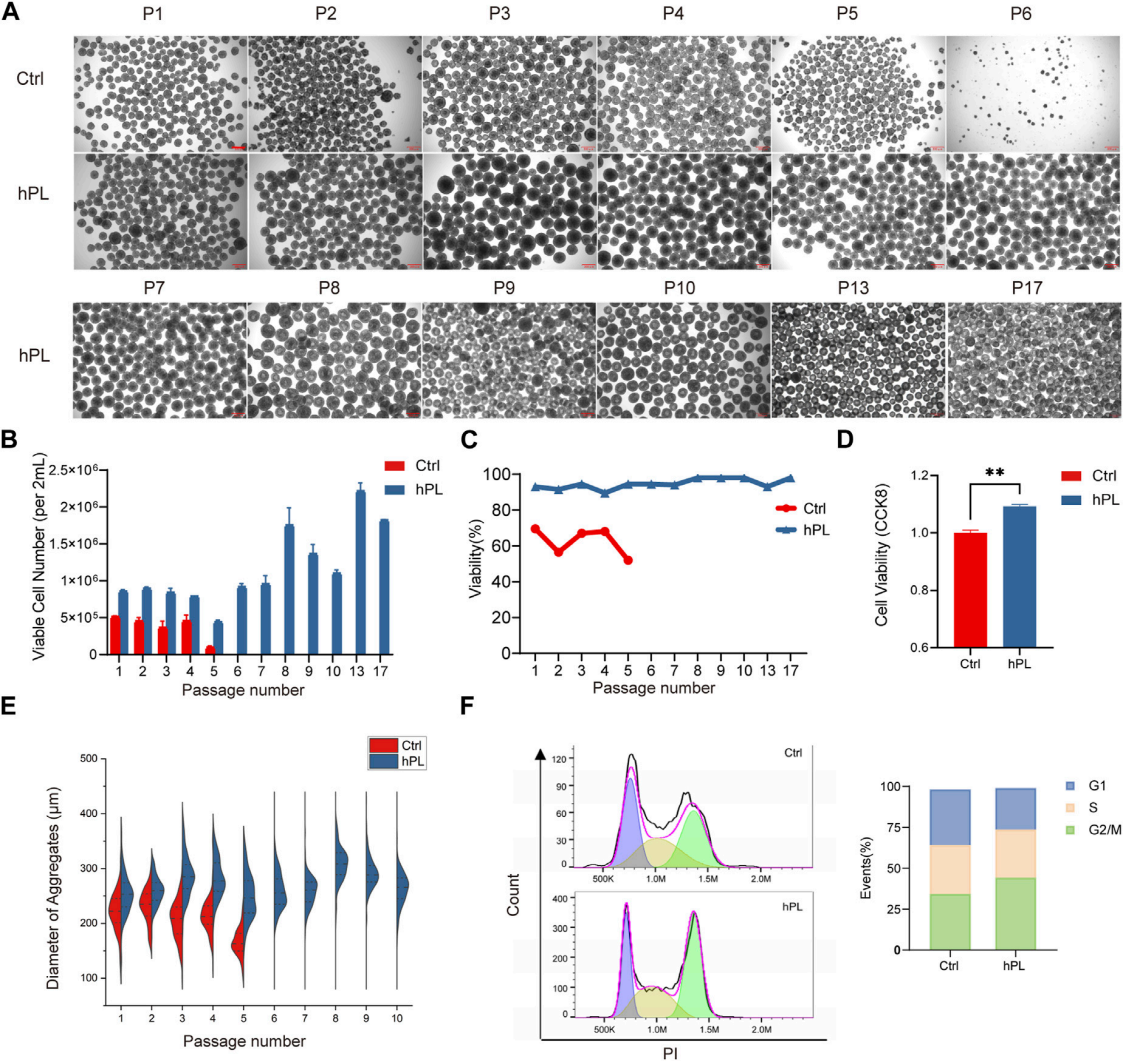
Enhanced viability and proliferation in hPL-supplemented 3D culture of hPSCs **(A)** Morphology of H1 cell aggregates under the 3D culture system at different generations. Scale bar = 300 μm. **(B)** Numbers of viable H1 cells at different generations during the 3D culture. **(C)** Cell viability of H1 cells at different generations determined via trypan blue staining. **(D)** Cell viability of H1 cells at the fourth generation measured by CCK8 assay, **p < 0.01. **(E)** Diameter of H1 cell aggregates at different generations. **(F)** Cell cycle analysis of H1 cells performed by flow cytometry. Ctrl and hPL represent H1 cells cultured without or with hPL, respectively.

To evaluate the effect of hPL on hPSC suspension culture, we supplemented hPL in the 3D culture system. We observed substantial proliferation benefit in hPL-treated H1 hESCs (Figures 1A,B) and PBMC-derived hiPSCs (Supplementary Figures S1A,B) throughout the 3D culture process, with as low as 0.3% hPL supplementation. Interestingly, while PBMC-derived hiPSCs showed normal expansion and passaging phenotype in both conditions (Supplementary Figures S1A–C), H1 hESCs grown in the absence of hPL exhibited noticeable morphological changes as early as the fifth generation (after being passaged five times in the 3D culture system), with a reduction in the diameter of cell aggregates (Figures 1A, E). By the sixth generation, no cell aggregates were formed in the hPL-free culture system. However, in the hPL-supplemented culture system, H1 cells were able to be passaged normally and form normal-size cell aggregates even up to the 20th generation (Figure 1A).

We also monitored the viable cell number and cell viability at each passage and found that the supplementation of hPL in H1 hESC culture increased the viable cell number by over 60% comparing to hPL-free group and maintained cell viability above 90%. In contrast, the viability of H1 cells cultured without hPL gradually declined to below 50% (Figures 1B,C). hiPSCs cultured with hPL also showed increased cell number although the viability is similar to the culture condition without hPL (Supplementary Figures S1B,C). In addition, hPL supplementary was also able to maintain higher than 90% viability for both hESCs and hiPSCs following the freeze/thaw cycle (Supplementary Figure S1D).

We compared the viability and cell cycle phase distribution between the hPL-treated H1 at the 10th generation and untreated H1 cells at fourth generation by CCK8 assay. Our findings revealed that hPL-treated H1 exhibited higher cell viability and an increased number of cells in the G2 and M phases, suggesting heightened cellular division activity (Figures 1D, F).

These results suggest that hPL supplement increased the proliferation and viability of hPSCs in the 3D culture system.

### 3.2 Preserved characteristics of hPSCs in hPL-supplemented 3D culture

**FIGURE 2:**
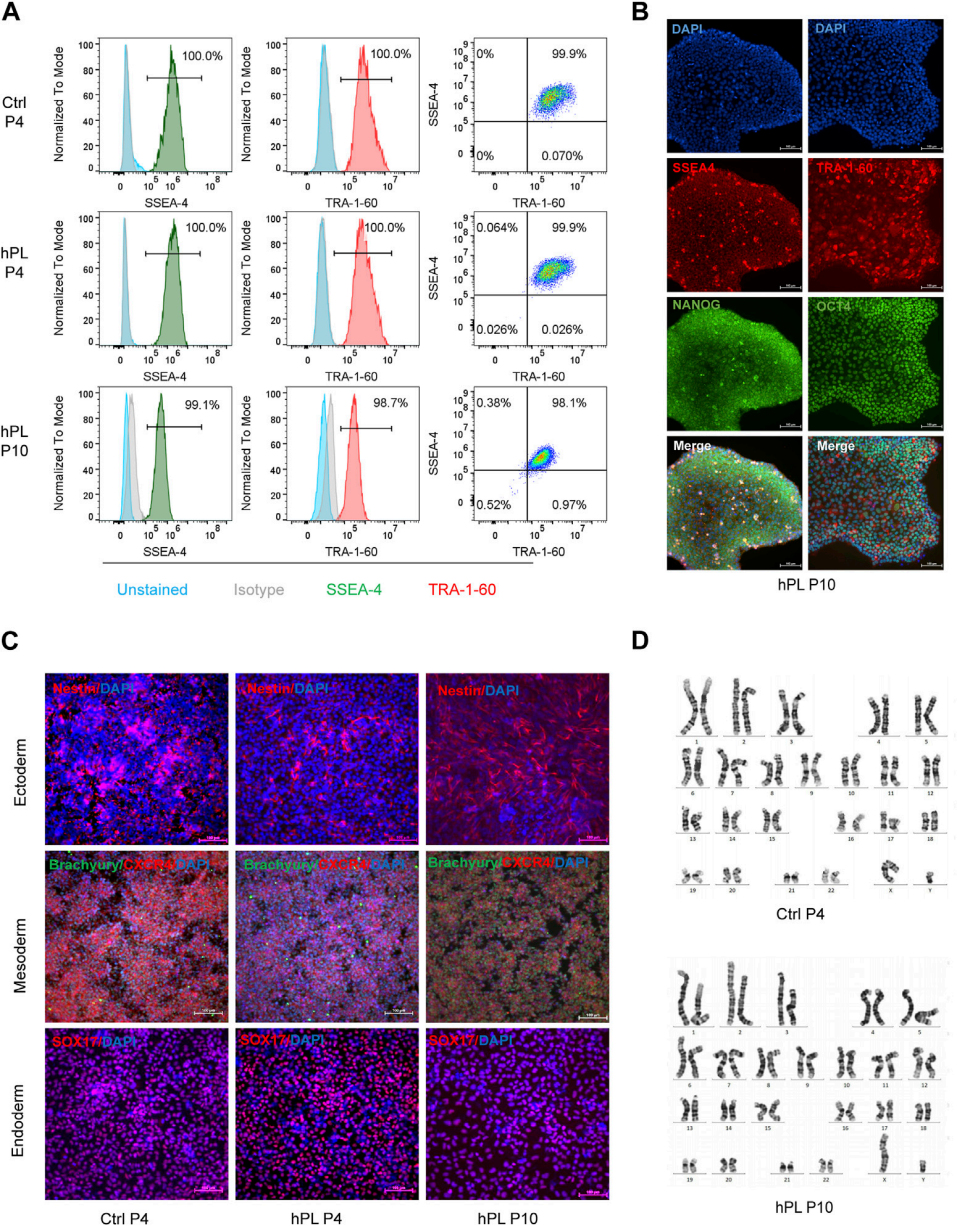
Preserved characteristics of hPSCs in hPL-supplemented 3D culture **(A)** Flow cytometry analysis of pluripotency markers SSEA4 and TRA-1-60 in hPL-treated (hPL) and untreated (Ctrl) H1 cells. **(B)** Immunofluorescence analysis of pluripotency markers SSEA4, TRA-1-60, NANOG and OCT-4 in hPL-treated H1 cells at 10th generation. Scale bar = 100 μm. **(C)** Trilineage differentiation potentials of hPL-treated and untreated H1 cells determined by STEMdiff Trilineage Differentiation kit. Scale bar = 100 μm. **(D)** Karyotype analysis of hPL-treated and untreated H1 cells. Ctrl and hPL represent H1 cells cultured without or with hPL, respectively.

To investigate whether hPL treatment affected the pluripotency of hPSCs, we performed flow cytometry analysis, immunofluorescence staining, and trilineage differentiation assay in hPL-treated H1 cells. Remarkably, more than 98% of hPL-treated H1 cells at the 10th passage stained positive for the cell surface markers SSEA-4 and TRA-1-60 and transcription factors NANOG and OCT4 (Figures 2A,B), suggesting that these cells maintained the undifferentiated state in aggregated culture. Furthermore, the trilineage differentiation assay demonstrated the hPL-treated H1 at the 10th generation retained the capability to differentiate into cells of all three germ layers (Figure 2C). In addition, these cells exhibited normal diploid karyotype even after extended culture (Figure 2D). Similar findings were observed for hiPSCs (Supplementary Figures S1F–G).

Taken together, these findings indicate that the effectiveness of hPL supplementation within the 3D culture environment in preserving the characteristic features of hPSCs, validating the viability of incorporating hPL as a supplement within the hPSC culture system.

### 3.3 Reduced intercellular variability and enhanced cell cycle regulation with hPL supplementation

In our pursuit of understanding the mechanism associated with hPL-driven proliferation in hPSCs within the 3D culture setup, we conducted RNA-seq analysis to capture the transcriptome profiles of hPL-treated and control H1 cells under the 3D culture system at the fourth generation.

**FIGURE 3:**
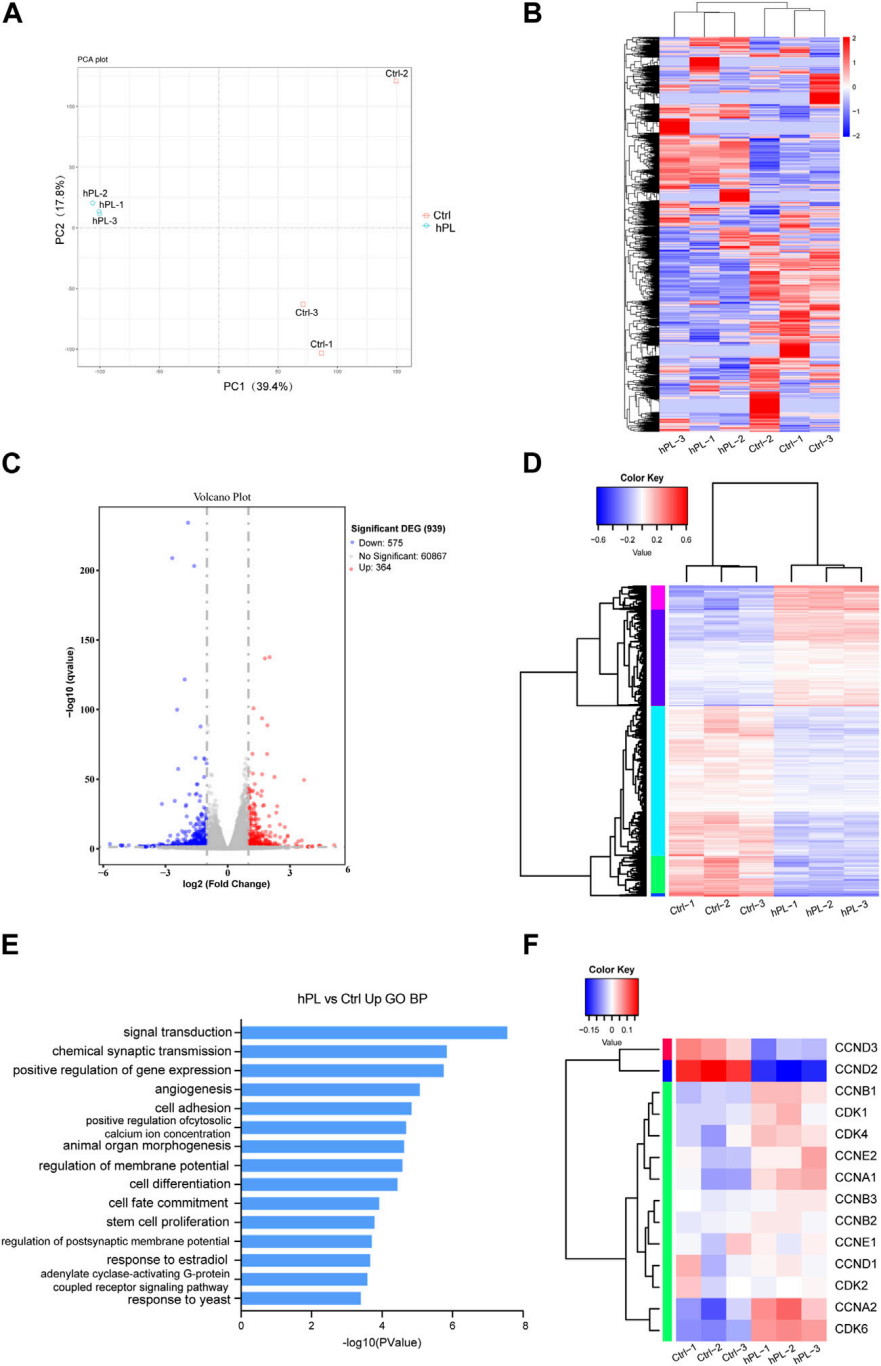
Transcriptomic analysis comparing hPL-treated and untreated H1 cells at the fourth generation **(A)** Principal Component Analysis (PCA) plot of hPL-treated and untreated H1 cells. **(B)** Heatmap of genes of the hPL-treated and untreated H1 cells. **(C)**Volcano plot of differentially expressed genes (DEGs) between hPL-treated and untreated H1 cells. **(D)** Heatmap of DEGS between the hPL-treated group and the untreated H1 cells. Three replicates were evaluated. **(E)** Gene Ontology (GO) Biological Process (BP) analysis of upregulated genes in hPL-treated H1 cells. **(F)** Analysis of expression of cyclins and cyclin-dependent kinases (CDKs) genes in hPL-treated and untreated H1 cells. Three replicates were evaluated. Ctrl and hPL represent H1 cells cultured without or with hPL, respectively.

Principal component analysis (PCA) revealed distinct clustering and discernible group formations for hPL-treated cells in the principal component space, whereas the distribution of hPL-free cells was more scattered (Figure 3A). This highlights that hPL supplementation mitigated variances across different cell batches.

Differences in the expression profile between untreated H1 and hPL-treated H1 are shown in the heat map of whole-genome gene expression (Figure 3B). Bioinformatics analysis revealed that 364 transcripts were upregulated (*p* < 0.05 and FC ≥ 2) and 575 transcripts were downregulated (*p* < 0.05 and FC ≤ 2) in hPL-treated hPSCs compared to hPL-free hPSCs (Figures 3C,D). Furthermore, we subjected the upregulated and downregulated genes to Gene Ontology (GO) analysis containing three sub-ontologies (BP, biological process; MF, molecular function; CC, cellular component) (Supplementary Figure S2). Interestingly, these upregulated genes were involved in several important biologic processes including positive regulation of gene expression and stem cell proliferation (Figure 3E). This finding aligns with our earlier experimental results, reaffirming that hPL bolsters hPSC proliferation within the 3D environment. Additionally, we found that a majority of genes associated with cyclins and cyclin-dependent kinases (CDKs) showed higher expression in hPL-treated cells. This observation suggests that hPL-treated cells under 3D culture system enhanced its cell cycle regulatory mechanisms, thereby enabling cells to undergo division and proliferation more efficiently, which is also consistent with our previous findings (Figure 3F).

Taken together, the analysis of transcriptome profile suggests that hPL-treated cells exhibit reduced intercellular variability. Additionally, the supplementation of hPL enhances cell cycle regulatory mechanisms, thereby promoting proliferation.

## 4 Discussion

Large-scale production of consistently high quality hPSCs are crucial to advance clinical applications of hPSC-based therapies. Several groups have developed the 3D aggregated culture systems for the expansion of hPSCs based on single-cell inoculation (Amit et al., 2010; Olmer et al., 2010; Wang et al., 2013). Simultaneously, the culture medium still remains a central focus to improve the efficiency and yield. Besides basal medium, additional medium components are usually supplied in the form of FBS or defined mixture such as KOSR or B27 supplement (Liu et al., 2006). However, with the advance in fields of cell and gene therapy, the xenogeneic supplements remain an obstacle for the translation of cell and gene therapy applications from bench to bedside. Therefore, alternative xeno-free medium is needed to meet the quality and quantity requirements in larger-scale hPSC production. In our effort to achieve this, we tested hPL, a xeno-free, serum-free supplement, in the culture of hPSCs within a small-scale 3D culture system. In our research, we assessed the effects of hPL supplementation on various aspects, including cell morphology, cell proliferation, viability, cell cycle, and the maintenance of hPSC characteristic properties.

In our current study, we observed an enhancement in both the cell proliferation capacity and cellular viability of hiPSC and H1 hESCs within the culture system supplemented with hPL. Furthermore, hPSCs maintained their characteristic properties in the hPL-supplemented 3D culture system. (Figure 1 and Supplementary Figure S1D). Interestingly, we observed, in the absence of hPL during the cultured process, H1 cells exhibited notable morphological alterations at the fifth passage and could not be expanded as aggregates (Figure 1A). However, within culture system supplemented with hPL, H1 cells exhibited normal passage behavior (Figure 1A). Since this alteration was not observed in hiPSCs, we believe that this phenomenon is likely attributed to the characteristics of the H1 cell line. H1 cell line has been reported to exhibit lowest proliferation efficiency compared to many other hPSC lines in a 3D culture system, which indicated it may have proliferation challenges (Lei and Schaffer, 2013). This improvement in H1 cells culture suggests that hPL possess the potential to improve the culture of various hPSC lines, even those that are more difficult to culture in the suspension conditions.

To explore the transcriptome changes related to hPL induced improvement in H1 cells, we performed RNA-Seq analysis (Figure 3 and Supplementary Figure S2). The analysis revealed significant alterations in the gene expression associated with cell cycle regulation, gene expression, and stem cell proliferation in hPL-treated cells. This could explain the mechanisms behind the enhancement in cell proliferation and viability. Notably, the upregulation of genes related to the cell cycle (Figure 3F) suggests that hPL-treated cells in the 3D culture system enhanced their cell cycle regulatory mechanisms, this enhancement likely contributes to more efficient cell division and proliferation. Moreover, PCA analysis revealed distinct clustering of hPL-treated cells within the principal component space, forming well-defined clusters. In contrast, untreated cells exhibited greater scatter (Figure 3A). This observation suggests that the cellular differences in the hPL-treated group are less pronounced, which aligns with our observed morphological changes and the distribution of cell aggregates diameters. This suggests that hPL has the potential to decrease inter-batch variability among cells when used as a supplement in the culture medium.

Our observations revealed enhancements of hPL supplement in cell proliferation capacity and cellular viability, along with a reduction in inter-batch variability. Additionally, in comparison to some other reported researches, we demonstrated advantages in certain aspects. In our hPL-supplemented system, even H1 cells could expand around 5-fold per passage. In comparison, the spinner flask culture system using E8 medium (Wang et al., 2013) exhibited an expansion of around 2.4 to 3.5-fold per passage, and with mTeSR (Chen et al., 2012), an average of 4.3-fold per passage expansion was reported. Additionally, hPL-treated cells maintained a higher proportion of pluripotent stem cell marker expression, comparing to those reported with TRA-1-60 expression at only 81% (Olmer et al., 2012). In comparison with other studies, the supplementation of hPL in our research has demonstrated advantages in terms of enhancing proliferation efficiency while maintaining pluripotency.

In summary, our study provides theoretical support for the application of hPL in 3D culture systems. As a xeno-free and serum-free supplement, hPL holds tremendous promise for advancing large-scale stem cell production, with potential implications for fields such as regenerative medicine. However, our experiments still have some limitations. Firstly, we conducted our experiments in a small-scale culture system and did not explore the potential benefits of hPL in larger bioreactors. Secondly, our study focused on a limited number of pluripotent cell lines. For future research, it would be more significant to explore the effects of hPL supplementation in larger culture systems employing expanded bioreactors. Additionally, a comprehensive assessment of a broader range of pluripotent cell lines, particularly those derived from patients or known to exhibit proliferation challenges, would promote the application of our findings in the field of pluripotent stem cell technology.

## Data Availability

The datasets presented in this study can be found in online repositories. The names of the repository/repositories and accession number(s) can be found below: NCBI SRA under PRJNA1019100.
