# Supplementary material for: Scalable expansion of human pluripotent stem cells under suspension culture condition with human platelet lysate supplementation
Source: Front Cell Dev Biol. 2023 Oct 12;11:1280682. doi: 10.3389/fcell.2023.1280682 (PMC10601454; doi:10.3389/fcell.2023.1280682)
Supplement: Supplementary file 1 [file Table1.DOCX]

Supplementary Material

# Supplementary Figures


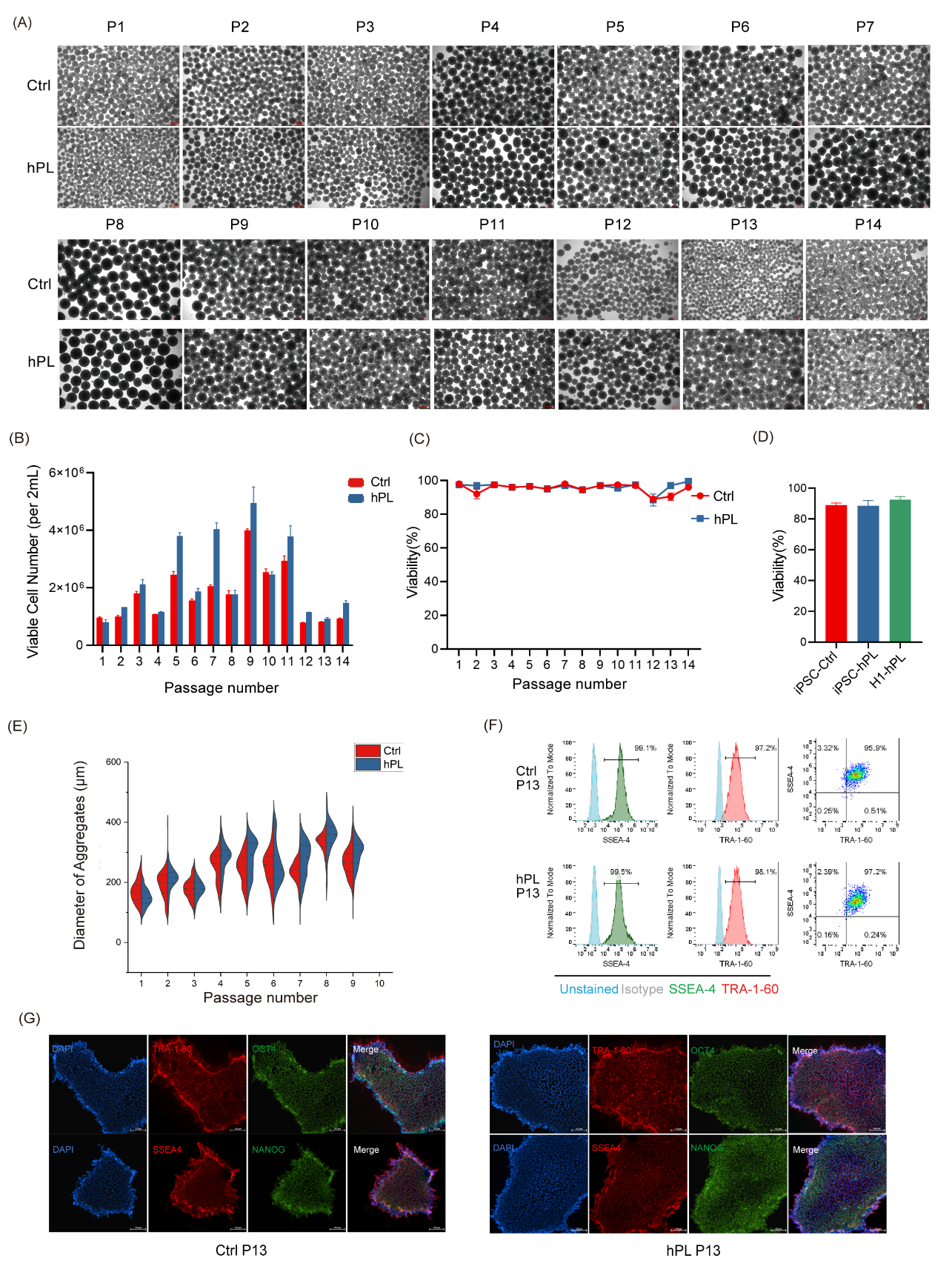


**Supplementary Figure 1. The characterization of hiPSCs in hPL-supplemented 3D culture**

(A) Morphology of hiPSC aggregates under the 3D culture system at different generations. Scale bar = 300μm. (B) Numbers of viable hiPSCs at different generations during the 3D culture. (C) Cell viability of hiPSC cells at different generations determined via trypan blue staining. (D)Cell viability of untreated hiPSC cells, hPL-treated hiPSC cells and hPL-treated H1cells following freeze/thaw cycle (E) Diameter of hiPSC aggregates at different generations. (F) Flow cytometry analysis of pluripotency markers SSEA4 and TRA-1-60 in untreated and hPL-treated hiPSCs at thirteenth generation. (G)Immunofluorescence analysis of pluripotency markers SSEA4, TRA-1-60, NANOG and OCT-4 in untreated and hPL-treated hiPSCs at thirteenth generation. Scale bar = 100μm.Ctrl and hPL represent cells cultured without or with hPL, respectively.


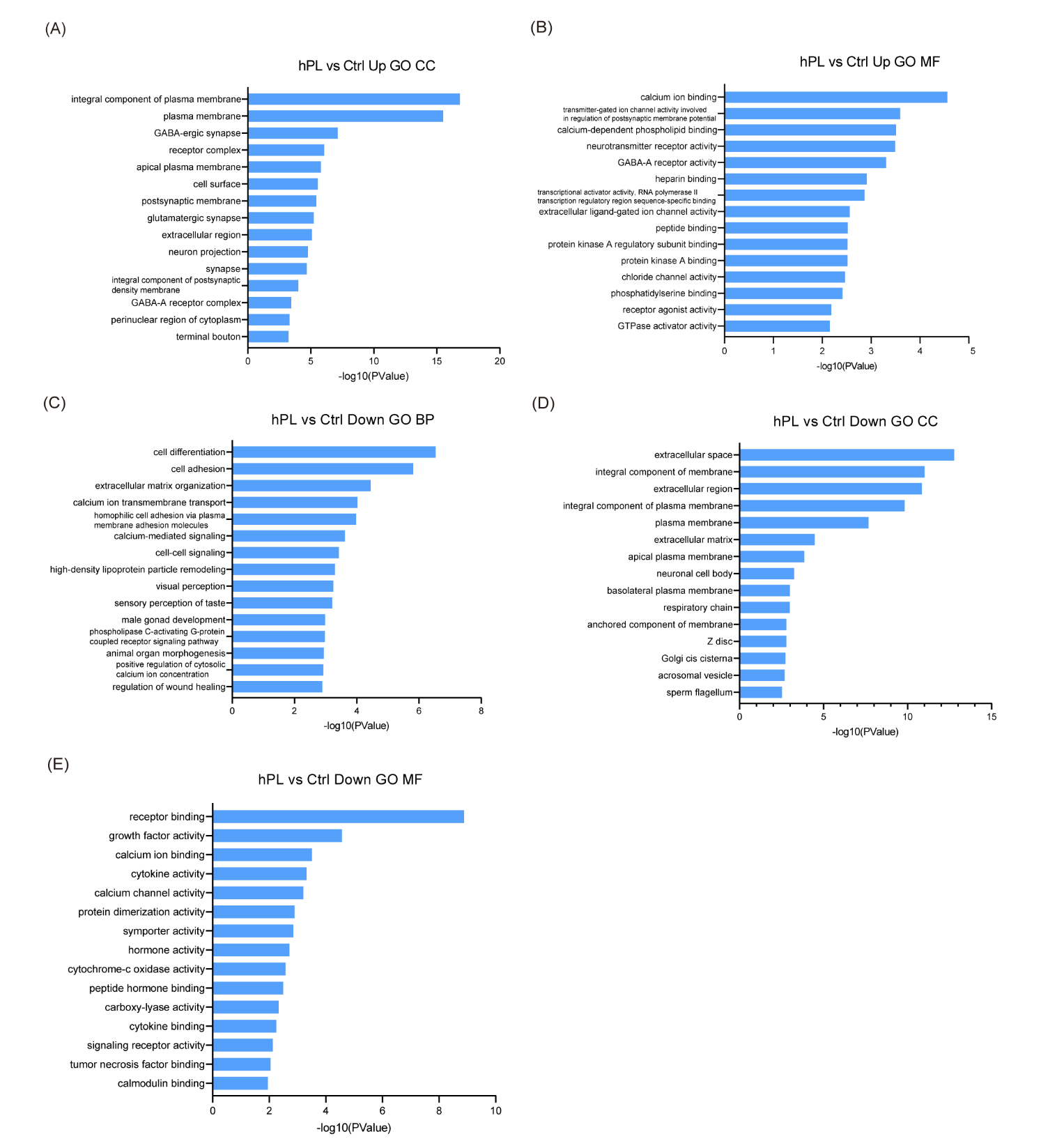


**Supplementary Figure 2. The GO analysis of upregulated and downregulated genes in hPL-treated H1 cells**

(A) GO Cell Component (CC) analysis of upregulated genes in hPL-treated H1 cells. (B) GO Molecular Function (MF) analysis of upregulated genes in hPL-treated H1 cells. (C) GO Biological Process (BP) analysis of downregulated genes in hPL-treated H1 cells. (D) GO CC analysis of downregulated genes in hPL-treated H1 cells. (E) GO MF analysis of downregulated genes in hPL-treated H1 cells. Ctrl and hPL represent H1 cells cultured without or with hPL, respectively.
